# Charge-Sensitive Optical Detection of Binding Kinetics between Phage-Displayed Peptide Ligands and Protein Targets

**DOI:** 10.3390/bios12060394

**Published:** 2022-06-08

**Authors:** Runli Liang, Yingnan Zhang, Guangzhong Ma, Shaopeng Wang

**Affiliations:** 1 Biodesign Center for Bioelectronics and Biosensors, Arizona State University, Tempe, AZ 85287, USA; rliang3@asu.edu (R.L.); guangzho@asu.edu (G.M.); 2 School of Electrical, Computer and Energy Engineering, Arizona State University, Tempe, AZ 85287, USA; 3 Department of Early Discovery Biochemistry, Genentech, South San Francisco, CA 94080, USA; zhang.yingnan@gene.com; 4 School of Biological and Health Systems Engineering, Arizona State University, Tempe, AZ 85287, USA

**Keywords:** charge-sensitive optical detection, binding kinetics, label-free, phage-display, peptide, protein

## Abstract

Phage display technology has been a powerful tool in peptide drug development. However, the supremacy of phage display-based peptide drug discovery is plagued by the follow-up process of peptides synthesis, which is costly and time consuming, but is necessary for the accurate measurement of binding kinetics in order to properly triage the best peptide leads during the affinity maturation stages. A sensitive technology is needed for directly measuring the binding kinetics of peptides on phages to reduce the time and cost of the entire process. Here, we show the capability of a charge-sensitive optical detection (CSOD) method for the direct quantification of binding kinetics of phage-displayed peptides to their target protein, using whole phages. We anticipate CSOD will contribute to streamline the process of phage display-based drug discovery.

## 1. Introduction

The global peptide therapeutics market is one of the fastest growing segments of the pharmaceutical industry right now, with the market valued at USD 20 billion in 2017 and expected to gain USD 50 billion in revenues by 2024 [1]. Compared to small molecule drugs, peptides have greater efficacy, selectivity, and specificity, with lower risks of systemic toxicity and complications. Compared to other biological drug candidates, such as antibodies and proteins, peptides are cheaper to manufacture and are smaller in molecule size so that it is easier for peptides to penetrate into organs or tumors. Peptides also offer higher activity per unit mass with greater stability compared to other biological drugs [2]. Phage display technology is one of the most widely used in vitro display technologies that offers high transformation efficiency with unique peptide clones. In addition, a higher number of copies can be displayed with phage than in other in vitro display technologies [3].

One bottleneck in phage display technology is the triage of hundreds of lead molecules for follow-up characterization. Currently, ELISA [4] is the only commonly used method for triaging clones while the displayed peptides are still on the phage’s surface. However, many peptides are displayed in multivalent form; thus, the ELISA signal reflects a combination of valency and affinity. Recent studies have found that multivalency and the spatial configuration of binding sites can significantly influence binding kinetics [5,6]. In this case, only qualitative data are provided. To find the best drug candidates from hundreds of positive peptides screened out of a phage-displayed peptide library, binding kinetics data between the peptide and the target protein is needed. In current practice, candidate peptides need to be custom synthesized for their binding kinetics measurement, and the process is costly and time-consuming. 

It can be challenging to use conventional label-free detection methods to measure phage-displayed peptide binding kinetics directly, such as surface plasmon resonance (SPR) [7] or bio-layer interferometry (BLI) [8]. SPR has been used to determine the affinity of phage-displayed peptides [9]; however, direct measurements of binding kinetics of the peptides displayed on the phage often proves to be difficult [10] due to the following reasons: (1) the huge mass of phage shifting the baseline out of detection range when the phage is loaded onto the sensor’s surface, (2) the surface density of the displayed peptides being limited by the expression level and the huge mass ratio between the phage and the peptide, and (3) the multivalent nature of phage-expressed peptides making the sensor response curves reflect the collective effect of multivalency binding kinetics instead of one-to-one first-order binding kinetics if loading the target protein onto the sensor’s surface. While electrochemical detection methods have been used in phage display technology, they focus on studying the capture of *E. coli* or other bacterial cells using phages [11,12,13]. Thus, it is still challenging to measure the binding kinetics of phage-displayed peptides or antibodies without the synthesis or purification of the peptides or antibodies. 

We have developed a charge-sensitive optical detection (CSOD) method that measures molecular interaction kinetics by detecting the binding-induced charge changes, which can overcome the mass sensitivity limit [14,15,16]. This unique feature provides CSOD with higher sensitivity than conventional mass sensitive label-free methods in measuring phage-target binding kinetics. In this work, we report an experiment scheme that combines CSOD with phage display technology to enable the rapid quantification of peptide binding kinetics directly on whole phages. 

## 2. Detection Principle

**Figure 1:**
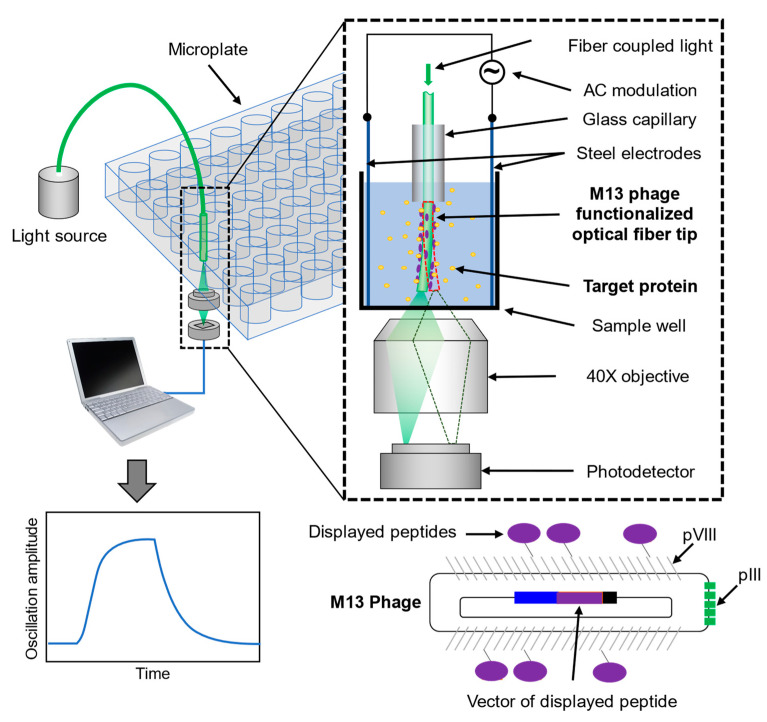
Schematic of charge-sensitive optical detection of binding kinetics between M13 phage-displayed peptides and the protein target. An optical fiber is inserted into a glass capillary and the fiber tip is dipped into a microplate well between a pair of steel electrodes. An alternating electric field is applied through the electrodes to drive the fiber tip into oscillation. The light coming out from the tip bottom is collected by a 40× objective and measured by a quadrant cell detector. For the binding kinetics measurement, the M13 phages with peptides displayed on pVIII coating proteins are immobilized on the fiber tip, and the tip is inserted into a well with target protein. Upon binding, the net charges of the M13 phages are changed and the oscillation amplitude of the fiber tip changes as well. By recording the oscillation change in real-time, the binding kinetics between the peptide, and the protein can be quantified.

A schematic of the CSOD system is shown in Figure 1. The principle of CSOD has been described in detail previously [14]. Briefly, a stripped glass optical fiber with a tip (length ~10 mm) functionalized with protein samples is dipped into a microplate well. If the solution well contains molecular ligands with charge or charged functional groups, the binding of the ligands to the proteins changes the net charge on the fiber’s surface. To monitor the charge change, an alternating electric field is applied perpendicular to the optical fiber and drives the fiber for oscillation. The oscillation amplitude is proportional to the surface charge of the optical fiber. The fiber oscillation amplitude is related to electric field strength, frequency, surface charge density, and fiber dimensions (length and diameter). The relationship is given by the following [14]:

(1) $$x=\frac{2\pi \left|\vec{E\left(\omega \right)}\right|\sigma rl}{\sqrt{{\left(k_{eff}-m_{eff}\omega^{2}\right)}^{2}+{\left(c\omega \right)}^{2}}}$$

where $\left|\vec{E\left(\omega \right)}\right|$ is the electric field strength applied to the fiber, *c* is the damping coefficient, and $k_{eff}$, $m_{eff}$, *r*, and *l* are the effective spring constant, mass, radius, and length of the optical fiber, respectively. The effective spring constant, $k_{eff}$, of the cylindrical optical fiber is given by the following:

(2) $$k_{eff}=\frac{3\pi Er^{4}}{4l^{3}}$$

where *E* is the Young’s modulus. The electric field applied is frequency dependent and is given by the following:

(3) $$\left|\vec{E\left(\omega \right)}\right|=\left|\vec{E_{0}}\right|\frac{R_{S}}{\sqrt{R_{S}^{2}+\frac{1}{{\left(\omega C_{eff}\right)}^{2}}}}$$

where $\left|\vec{E_{0}}\right|$ is the input electric field between the electrodes, and *R_s_* and *C_eff_* are the solution resistance and effective interfacial capacitance of electrode/solution interface, respectively. Equations (1)–(3) show that the oscillation amplitude is both frequency and fiber dimension-dependent. In our previous studies, we typically use fibers with a small diameter (around 15 μm), and the optimal frequency where fiber oscillates at maximum amplitude is usually low (below 60 Hz), which is suitable for detections with a CCD camera with a limited frame rate [14,15]. Since CSOD detects the mechanical motion of the fiber tip, the oscillation signal is sensitive to environmental mechanical noise. From a noise spectrum analysis shown in Figure S1 where no electric field was added, the noise levels are much higher in the low frequency region, which are mostly from environmental and system mechanical noises that are hard to isolate at low frequencies. In this study, we used larger diameter fibers (around 120 μm) with higher resonance frequency (Figure S2) and achieved four times improved signal-to-noise ratio (Figure S3). Using large fiber also significantly reduced the usage time of the toxic hydrofluoric acid for fiber etching from ~30 min to ~30 s, with the possibility to eliminate this step in the future. For sustained recordings of the high frequency signal, we replaced the CCD camera with a high-speed photo detector, which reduced the data size by three orders of magnitude due to the photodetector recording only differential and sum signals, rather than the image stacks recorded by the camera. These implementations dramatically improved the performance of CSOD technology and prepared it for future developments of high-throughput detection.

**Figure 2:**
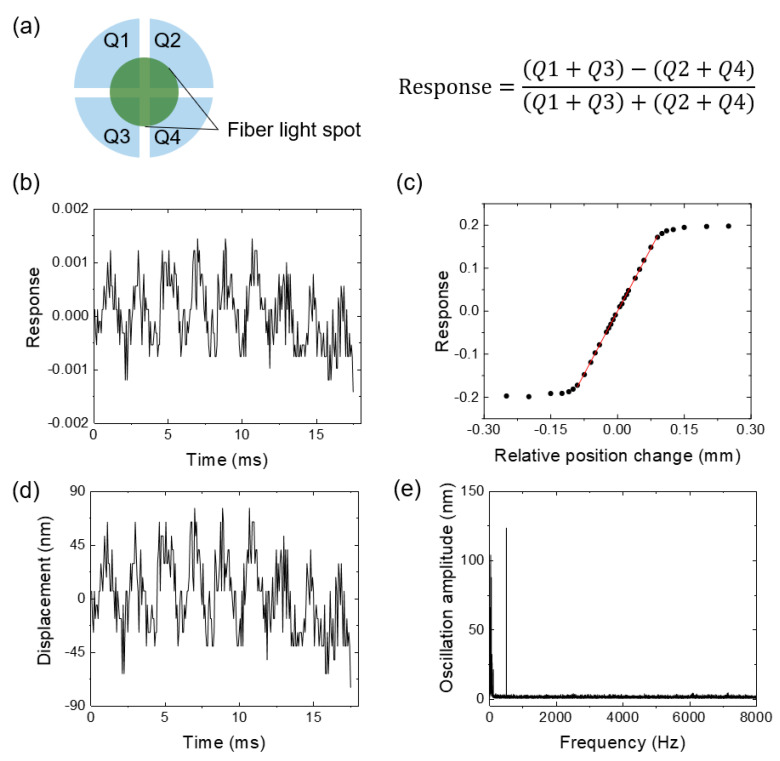
CSOD data processing workflow. (**a**) Schematic shows the light out of the fiber tip projected on the center of the quadrant cell detector, where the sensor response is calculated with the equation shown on the right. *Q*1 to *Q*4 are the signals collected by the quadrant cell detector. (**b**) Representative measured detector response plotted against time with a sampling rate of 16.000 data points per second. (**c**) The calibration curve and linear region of the quadrant cell detector. (**d**) The calibrated fiber oscillation amplitudes over time. (**e**) The fast Fourier transform (FFT) result of the fiber oscillation amplitude in 1 s, where the peak at 511 Hz clearly shows the fiber vibrates at the set frequency.

To measure the oscillation amplitude, light is coupled into the fiber and received by a quadra-cell position-sensitive photodetector placed under the fiber tip (Figure 1). The oscillation of the fiber changes the position of the outcoming light spot on the detector and is recorded in real-time using a differential algorithm (Figure 2a). The electric output of the detector (Figure 2b) is then converted to the relative position change in nanometers using a calibration curve (Figure 2c, see also Methods), and the oscillation profile of the fiber tip can be obtained (Figure 2d). Fast Fourier transform (FFT) is applied to the oscillation profile every one second. The FFT spectrum shows a pronounced peak at 511 Hz, which is the frequency being applied, and the height of the peak represents the oscillation amplitude of the fiber tip (Figure 2e).

## 3. Materials and Methods

### 3.1. Materials

Multimode optical fibers (125 μm in diameter, FG105UCA, Thorlabs, Newton, NJ, USA) were purchased from Thorlabs, Inc. Phosphate-buffered saline (PBS) was purchased from Mediatech Inc., Manassas, VA, USA. The drug target proprotein convertase subtilisin/kexin type 9 (PCSK9) was provided by Dr. Daniel Kirchhofer at Genentech [17]. N-hydroxysulfosuccinimide (NHS), N-(3-dimethylaminopropyl)-N′-ethylcarbodiimide hydrochloride (EDC), and O-(2-carboxyethyl)-O′-(2-mercaptoethyl) heptaethylene glycol (SH-PEG8-COOH) were purchased from Sigma-Aldrich. Streptavidin, methyl-PEG4-thiol (MT(PEG)4) and SuperBlock blocking buffer were purchased from Thermo Fisher Scientific. Deionized (DI) water with a resistivity of 18.2 MΩ·cm filtered through a 0.45 μm filter was used in all experiments. Other chemicals were purchased from Sigma-Aldrich.

### 3.2. Generation of M13 Phage Displayed Peptides

Five different M13 phage samples were generated and used as listed in Table 1. The peptides were fused to the N-terminus of M13 major coat protein (pVIII) utilizing two different types of secretion signal peptides (Table 1) [18], with or without the leading sequence of Ser-Gly (SG) extension at the N-terminus of the peptide. Peptides with double mutations of F3A:W6A served as negative controls. The resulting constructs were transformed to *E. coli.* XL1 blue and single colonies grown in 1 mL 2YT supplemented with 50 μg/mL carbenicillin, 10 μg/mL tetracycline, and M13 KO7 helper phage at 37 °C for 2 h. After the addition of kanamycin (25 μg/mL) and a 6 h incubation at 37 °C, the culture was transferred to 30 mL 2YT supplemented with 50 μg/mL carbenicillin and 25 μg/mL kanamycin and grown at 37 °C overnight. Phages were harvested and purified using the standard protocol [4].

### 3.3. Charge-Sensitive Optical Detection Setup

An inverted microscope (Olympus IX-70) with a 40X objective and a quadra-cell position-sensitive photodetector (PDQ80A, Thorlabs) placed at the side camera port were used for CSOD detection. A 96-well microplate with a pair of steel electrodes (1 cm × 0.6 cm, 0.8 cm distance) inside the wells were mounted on a motorized microscope stage (BioPrecision2, Ludl Electronic Products LTD., Hawthorne, NY, USA). Light from a laser (532 nm, 20 mW, Prometheus, Coherent, Santa Clara, CA, USA) was coupled into the fiber via an objective lens (2X, NA 0.06, Olympus). To move the fiber probe between wells, the fiber was clamped to a motorized arm (A-LSQ075B, Zaber, Vancouver, BC, CA) via a post-mounted fiber clamp (T711-250, Thorlabs) to lift the fiber up and down. A sinusoidal potential was applied with a function generator (33521A, Agilent). A USB data acquisition card (USB-6228, National Instruments) was used to record the output of the photodetector.

### 3.4. Surface Functionalization

The tip (about 1 cm) of an optical fiber thread (about 20 cm) was first soaked in acetone for 1 min and then rinsed with DI water and dried. The polymer coating layer on the optical fiber was then stripped off with an optical fiber stripper. The bare fiber was etched by soaking it in 47% hydrofluoric acid for 30 s for a diameter of ~120 μm. The etched fiber was later rinsed with DI water to wash off the hydrofluoric acid and then blow-dried with nitrogen. The tip was cut to about 9 mm long. Before functionalization, the optical fiber was cleaned with oxygen plasma for 3 min.

The etched fiber was soaked in (3-glycidyloxypropyl)trimethoxysilane (epoxy) solution (2.5% volume percentage of epoxy in isopropanol) for 1 h for surface functionalization. The fiber was then rinsed with a PBS buffer. The epoxy modified fiber was soaked in an M13 phage sample solution (10^7^ pfu/mL in 1X PBS) for 1 h.

### 3.5. Calibration Curve

The optical fiber was mounted onto a piezo actuator (PAS005, Thorlabs) via a fiber clamp (T711-250, Thorlabs) for precise fiber movement. As shown in Figure 2a, the total quadrant cell response was calculated by the following:

(4) $$Response=\frac{\left(Q_{1}+Q_{3}\right)-\left(Q_{2}+Q_{4}\right)}{\left(Q_{1}+Q_{3}\right)+\left(Q_{2}+Q_{4}\right)},\;\;\;\;\;\;\;\;\;\;\;\;\;\;\;\;$$

where *Q*_1_, *Q*_2_, *Q*_3_, and *Q*_4_ are the responses from each cell. The quadrant cell is equivalent to a bi-cell detector in this configuration. The total quadrant cell response was then plotted against the fiber moving distance provided by the piezo actuator as the calibration curve shown in Figure 2c.

### 3.6. SPR Measurement

The SPR sensor chip was fabricated by coating 1.5 nm Cr and then 47 nm Au on cover glass (no.1 VWR) using an e-beam evaporator. Prior to surface functionalization, the gold surface was cleaned with ethanol and DI water twice, dried by N_2_, and annealed by H_2_ flame. The gold chip was immersed in a mixture of 0.2 mM SH-PEG8-COOH and 0.2 mM MT(PEG)4 overnight. Then, -COOH was activated using NHS/EDC chemistry (50 mM NHS and 200 mM EDC) for 20 min and incubated with 0.1 mg/mL streptavidin in PBS for 30 min to allow immobilization. The remaining unreacted sites were quenched with 10 mM ethanolamine and the nonspecific binding sites were blocked with SuperBlock for 10 min. Next, the streptavidin-coated chip was incubated in 0.5 mM biotinylated pep2-8 for 30 min to capture the peptide on the surface. The functionalized chip was finally rinsed with PBS before the measurement.

## 4. Results and Discussion

**Figure 3:**
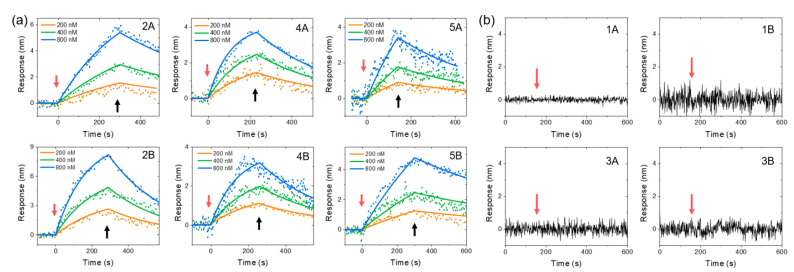
CSOD measurement results of PCSK9 binding to different peptides displayed on the M13 phage surface. Sample IDs are marked on the top right corner of each plot and the details of the samples are listed in Table 2. (**a**) Binding curves for different peptides. The fiber probes were switched from a well containing a buffer to a well containing PCSK9 at 0 s for the measurement of the association process (red arrow) and then switched back to the buffer well at the time indicated by the black arrow for the measurement of the dissociation process. (**b**) CSOD measurement results of PCSK9 binding to negative samples. The red arrows indicate switching the fiber probe from the buffer well to a well with 1000 nM PCSK9 solution.

Proprotein convertase subtilisin/kexin type 9 (PCSK9) regulates plasma low-density lipoprotein (LDL) cholesterol levels by degrading liver LDL receptors [19,20,21]. PCSK9 acts by binding to the EGF(A) domain of LDL receptor on the cell surface via its catalytic domain [22]. Previously, a peptide, designated as pep2-8, that inhibits PCSK9 binding to the EGF(A) domain of LDL receptor was reported to be effective in lowering LDL cholesterol [23]. The binding kinetics between PCSK9 protein and phage-displayed pep2-8 in various formats listed in Table 1 were measured using CSOD technology. Pep2-8-displayed M13 phage samples were functionalized to the fiber probe’s surface via epoxy coupling. The binding of different concentrations of PCSK9 proteins to the functionalized probes was studied in a 40-time diluted PBS buffer. For each measurement, a baseline was first established by dipping the functionalized fiber tip into a microplate well with a buffer; then, the fiber was switched to another well containing PCSK9 protein solution to measure the association curve. Finally, the fiber was moved back to the buffer well and the dissociation process was recorded. The measured binding kinetics curves are shown in Figure 3a. By fitting the kinetic curves at different concentrations globally with first-order kinetics using Scrubber v.2.0c, the association rate constants $\left(k_{a}\right)$, dissociation rate constants $\left(k_{d}\right)$, and equilibrium constants $\left(K_{D}\right)$ for all samples are summarized in Table 2. To confirm the binding specificity, negative controls with mutation of two critical amino acids involved in binding were included. Sample groups 1 and 3 are negative controls with displayed peptides that do not bind to PCSK9 protein. As shown in Figure 3b, PCSK9 has no measurable responses to any negative sample. 

**Figure 4:**
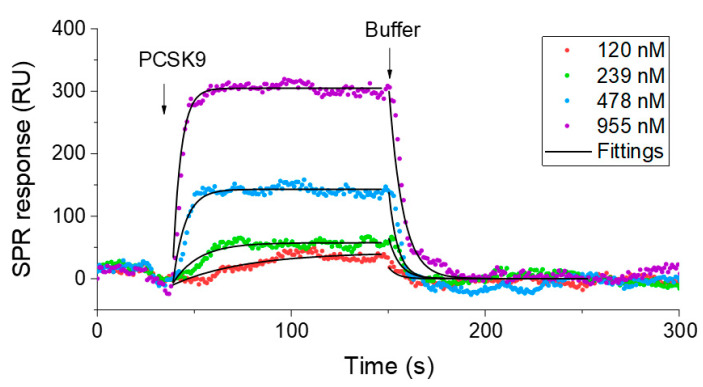
SPR measurement results of PCSK9 binding to synthesized pep2-8. The synthesized peptide was immobilized to a streptavidin-coated gold surface. Different concentrations of PCSK9 were flowed to the surface to measure the binding kinetics. By fitting the data obtained with different concentrations, the average and standard deviation for *k_a_*, *k_d_*, and *K_D_* were determined to be (3.88 ± 3.26) × 10^5^ M^−1^s^−1^, (1.73 ± 0.25) × 10^−1^ s^−1^, and 0.985 ± 0.724 µM, respectively.

As shown in Table 2, consistent affinity results (20% variation in *K_D_*) between sample groups A and B were obtained by CSOD, which are replicates of the same peptide construction, indicating good reproducibility of the method. However, variations in kinetic constants among the different constructed pep2-8 peptide sample group were observed, with twofold affinity variation between phage clones with two different secretion signal peptides (Sample #2 vs. #4), as well as a twofold affinity drop with SG extension (Sample #4 vs. #5) at the N-terminus. These variations in binding constants imply that the peptide’s construction methods may affect the binding kinetics of the peptide to the target protein. Nevertheless, the CSOD-measured equilibrium constants *K_D_* are in the range of 0.370–1.04 µM among the positive peptide sample groups, which are consistent with the previously reported *K*_D_ value of 0.66 µM between synthesized pep2-8 and PCSK9 determined by biolayer interferometry and the IC_50_ value of 0.81 µM measured by competition ELISA, where the binding of biotinylated PCSK9 to plate-coated LDL receptors is competed off by synthesized pep2-8 [23]. To further confirm our results, we performed an additional measurement of synthesized pep2-8—PCSK9 binding using SPR (Figure 4), which showed that *K_D_* was 0.985 ± 0.724 µM and is also consistent with the above results. Note that the *k_a_* and *k_d_* are different from those determined by CSOD, which implies that the microenvironment of peptides displayed on the phage may have an impact on ligand-binding kinetics.

Unlike reference technologies that can only measure synthesized peptides, CSOD directly measures peptides displayed in very high copy numbers on the phage. Having a high copy number is known to cause the multivalent binding of the target protein and leads to inaccurate binding kinetics, but CSOD can correctly measure the binding kinetics between the peptide and the protein ligand regardless of the copy number of peptides displayed on the phage, because the phage is immobilized on the sensing probe, and the monomer protein ligands are provided in the solution phase. On the other hand, it is impossible for reference technologies to employ the same strategy due to the huge mass of the phage. In addition, CSOD has a wide affinity range from several hundreds of pM to µM [12,13] and the detection throughput can be further improved up to 48 binding pairs per microplate. We hope that these unique features of CSOD will make it a useful tool for studying the binding of PCSK9-binding peptides.

## 5. Conclusions

We demonstrated the direct quantification of binding kinetics between phage-displayed peptides and the corresponding protein target by CSOD technology. We have shown that CSOD can be used to distinguish different binding kinetics of different peptides displayed by phage display technology. The CSOD-measured dissociation constants were validated by the literature reported values measured with synthetic peptides. These results show that CSOD can be incorporated into a diversity display pipeline for rapidly triaging the best peptide leads during affinity maturation stages and can increase the productivity of library screening. 

## Figures and Tables

**Table 1:** M13 Phage samples and descriptions.

			Displayed Peptide Sequence															
Sample ID	Sample Name	Note			1	2	3	4	5	6	7	8	9	10	11	12	13	Signal Peptide
1A, 1B	no SG pep2-8AA-2202	negative control			T	V	A	T	S	A	E	E	Y	L	D	W	V	MKIKTGARILALSALTTMMFSASAYA
2A, 2B	no SG pep2-8-2202	positive			T	V	F	T	S	W	E	E	Y	L	D	W	V	MKIKTGARILALSALTTMMFSASAYA
3A, 3B	no SG pep2-8AA-2478	negative control			T	V	A	T	S	A	E	E	Y	L	D	W	V	MKKNIAFLLASMFVFSIATNAYA
4A, 4B	no SG pep2-8-2478	positive			T	V	F	T	S	W	E	E	Y	L	D	W	V	MKKNIAFLLASMFVFSIATNAYA
5A, 5B	SG pep2-8-2478	positive	S	G	T	V	F	T	S	W	E	E	Y	L	D	W	V	MKKNIAFLLASMFVFSIATNAYA

Note: (1) pep2-8 is the ligand that binds to PCSK9; pep2-8AA is the peptide with two critical positions that are involved in binding mutated relative to Ala (marked in red color), with no binding to PCSK9; (2) 2202 and 2478, represented phagemids with two different signal peptides, may have different peptide display level on phage; (3) no SG/SG indicating without or with leading sequence of SG before the start of pep2-8 (marked in blue color). This may also affect the peptide display level. (4) Sample groups A and B are identical replicates prepared separately.

**Table 2:** CSOD measured kinetic and equilibrium constants of PCSK9 binding to phage surface-displayed peptides. The constants were determined by global fitting the sensor response curves at different PCSK9 concentrations relative to first-order kinetics.

Sample	Description	$k_{a}\;\left(M^{-1}\cdot s^{-1}\right)$	$k_{d}\;\left(s^{-1}\right)$	$K_{D}\;\left(\mu M\right)$
2A	no SG pep2-8-2202	$\left(1.66\pm 0.01\right)\times 10^{3}$	$\left(1.73\pm 0.03\right)\times 10^{-3}$	1.04±0.02
2B	no SG pep2-8-2202	$\left(3.44\pm 0.01\right)\times 10^{3}$	$\left(2.91\pm 0.02\right)\times 10^{-3}$	0.846±0.008
4A	no SG pep2-8-2478	$\left(5.05\pm 0.01\right)\times 10^{3}$	$\left(2.83\pm 0.02\right)\times 10^{-3}$	0.560±0.005
4B	no SG pep2-8-2478	$\left(3.19\pm 0.01\right)\times 10^{3}$	$\left(1.98\pm 0.03\right)\times 10^{-3}$	0.621±0.011
5A	SG pep2-8-2478	$\left(4.20\pm 0.40\right)\times 10^{3}$	$\left(1.54\pm 0.05\right)\times 10^{-3}$	0.370±0.047
5B	SG pep2-8-2478	$\left(3.13\pm 0.01\right)\times 10^{3}$	$\left(1.19\pm 0.02\right)\times 10^{-3}$	0.380±0.008

## Data Availability

The data for Figures is provided in Source Data.xlsx as a Supplementary Material of this article.

## Supplementary Materials

The following supporting information can be downloaded at: https://www.mdpi.com/article/10.3390/bios12060394/s1, Figure S1: CSOD system noise analysis; Figure S2: Frequency dependency of the oscillation amplitude of a large diameter fiber; Figure S3: Comparison of CSOD signal longterm stability for small and large diameter fibers. Source Data.xlsx is source data for Figure 2, Figure 3 and Figure 4.
