# Supplementary material for: Charge-Sensitive Optical Detection of Binding Kinetics between Phage-Displayed Peptide Ligands and Protein Targets
Source: Biosensors (Basel). 2022 Jun 8;12(6):394. doi: 10.3390/bios12060394 (PMC9221260; doi:10.3390/bios12060394)
Supplement: Supplementary file 1 [file biosensors-12-00394-s001.zip › biosensors-1740146-supplementary.pdf]

## Supporting Information

# Charge-Sensitive Optical Detection of Binding Kinetics between Phage-Displayed Peptide Ligands and Protein Targets

**Runli Liang [1,2], Yingnan Zhang [3], Guangzhong Ma [1] and Shaopeng Wang [1,4,*]**

[1] Biodesign Center for Bioelectronics and Biosensors, Arizona State University, Tempe, AZ 85287, USA; rliang3@asu.edu (R.L.); guangzho@asu.edu (G.M.)
[2] School of Electrical, Computer and Energy Engineering, Arizona State University, Tempe, AZ 85287, USA
[3] Department of Early Discovery Biochemistry, Genentech, South San Francisco, CA 94080, USA;
zhang.yingnan@gene.com
[4] School of Biological and Health Systems Engineering, Arizona State University, Tempe, AZ 85287, USA
* Correspondence: shaopeng.wang@asu.edu

## S1. Noise spectrum of CSOD system

The noise of the CSOD system was analyzed by using a large-diameter fiber. The fiber was dipped into a PBS solution similar to the experimental conditions. No electric field was added. Images of fiber tip were collected at 2000 frames per second using a fast CMOS camera (Mikrotron EoSens 3CXP).

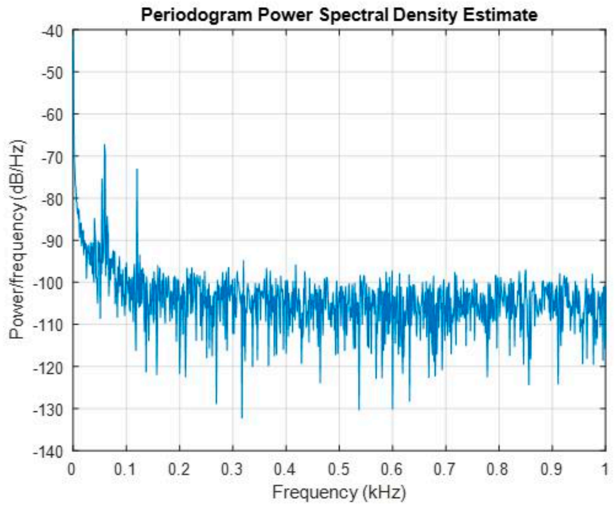


**Figure S1. CSOD system noise analysis.** Frequency analysis of a fiber tip imaged at 2000 frames per second without an electric field. Fiber diameter: 125 µm; length: 10 mm.

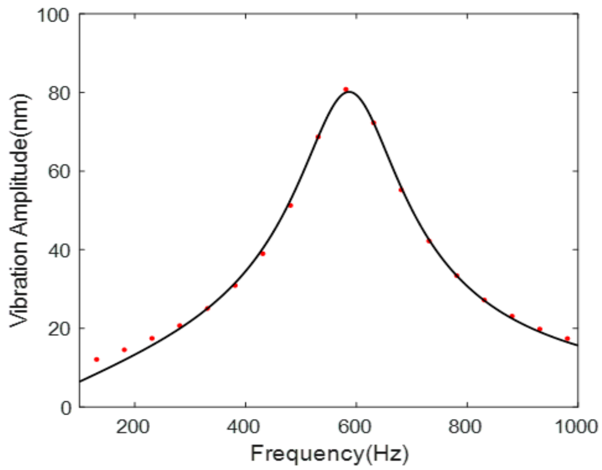


**Figure S2. Frequency dependency of the oscillation amplitude of a large diameter fiber.** The red dots are experimental data, and the solid line is calculated from Equation 1. Fiber diameter: 105.6 µm; length: 9.8 µm. Buffer: 40 times diluted 1X PBS buffer.

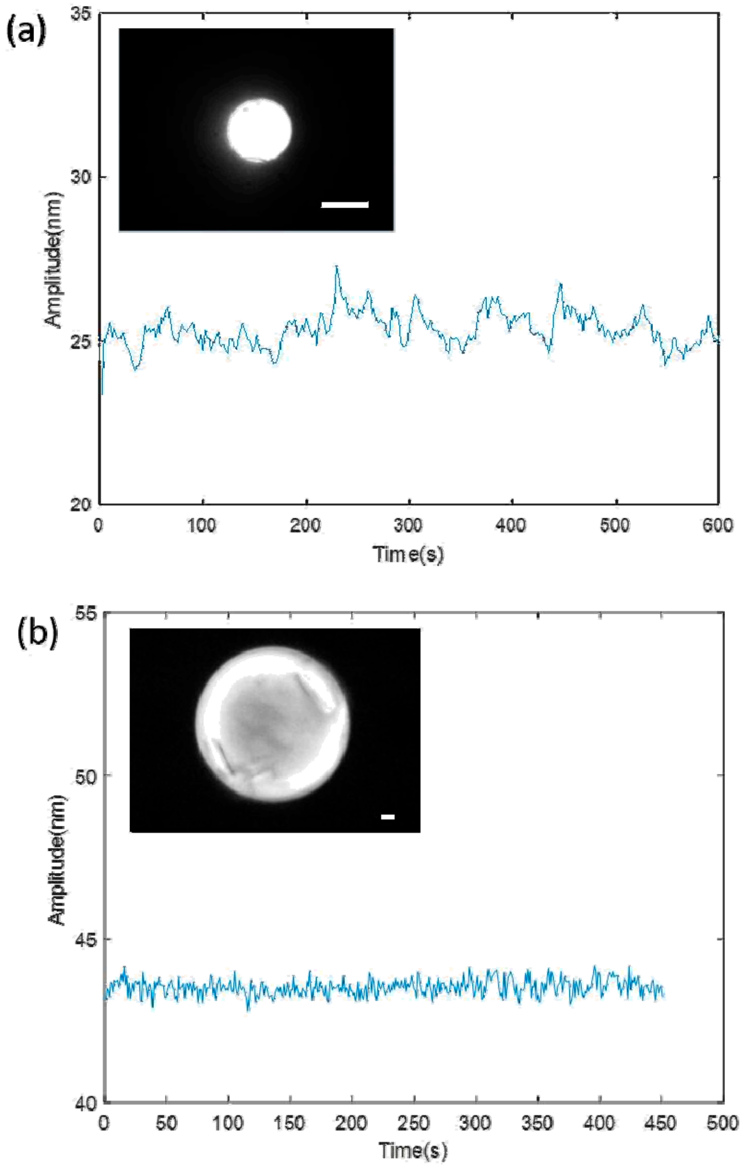


**Figure S3. Comparison of CSOD signal long-term stability for small and large diameter fibers.** (a) Small fiber with diameter 13.8 µm and length 9.0 mm oscillates at 35 Hz and recorded at 100 fps, 2 second per FFT amplitude data point. Average signal: 24.96 nm; noise (standard deviation) = 0.51nm. (b) Large fiber with diameter 112.0 µm and length 10.0 mm oscillates at 481 Hz, recorded at 2000 fps, 1 second per FFT amplitude data point. Average signal: 44.33 nm; noise (standard deviation) = 0.25 nm. The scale bar is 10 µm.
